# SERV-India instrument for measuring service quality in healthcare: development and pilot testing

**DOI:** 10.1186/s12913-026-14936-x

**Published:** 2026-06-17

**Authors:** Seep Sonali, Shweta Shenoy

**Affiliations:** MYAS-GNDU Department of Sports Sciences and Medicine, Guru Nanak Dev University, Amritsar, Punjab 143001 India

**Keywords:** Service quality (SQ), SERVQUAL, SERV-INDIA, Baksheesh, Sifarish, Healthcare service quality (HCSQ)

## Abstract

**Aim:**

The aim of the present study was to develop a viable instrument for measuring service quality (SQ) in the healthcare sector, including a new pool of factors identified that largely influence service quality primarily in the Indian healthcare sector (SERV-INDIA).

**Methods:**

An item pool was created by utilizing the SERVQUAL questionnaire as a generic model and an expert panel evaluated the items to assess the content validity index and ratio. The reliability, including internal consistency and reproducibility, was examined via Cronbach’s alpha coefficient and the Pearson’s r correlation. In addition, a known-group validity test was performed to evaluate how well the questionnaire discriminates the service quality satisfaction index between patients’ expectations and patients’ perceptions and among individuals who are receiving treatment under cash and insurance both public and private during present hospitalization. Also construct validity was performed using Confirmatory factor analysis (CFA).

**Results:**

A total of one hundred and three participants were studied for pilot testing. The mean age group of the participants was 30–40 years; almost 50% had studied until high school in the context of formal education. Overall, 7% of the participants were low, 19% were marginal, and 70% had an adequate service quality satisfaction index. The internal consistency, as measured by Cronbach’s alpha, was found to be 0.956 for Part 1 and 0.965 for Part 2 of the questionnaire. For known-group validity, participants who took treatment under cash during hospitalization had significantly higher satisfaction scores (*P* < 0.001) than patients with insurance, both public and private. In addition to this, the association between service quality satisfaction scores and Sifarish was also significant (*P* < 0.001). The CFA demonstrated acceptable fit on multiple indices for both parts of the questionnaire (CFI = 0.876 & 0.804 and SRMR = 0.036 & 0.065).

**Conclusion:**

SERV-INDIA is a valid and reliable measure of service quality in the healthcare sector, with newly introduced significant indicators like Mode of payment including Baksheesh and Sifarish.

**Supplementary Information:**

The online version contains supplementary material available at 10.1186/s12913-026-14936-x.

## Introduction

India is attempting to transform its health sector while going through a triple transition that is economic, demographic, and epidemiological. This presents both opportunities and challenges for the country in its efforts to modernize the health sector [1]. Indian health service users confront significant obstacles pertaining to physical service accessibility, cost-effectiveness, and substandard care quality. While challenges in physical access and adequate availability of quality health services act as major deterrents to people in accessing public health facilities, quality and cost tend to be more salient in the private sector. In the private sector, where providers involved in health-care delivery are quite diverse, compromises on the quality of services provided, including irrational, unnecessary and expensive interventions, are quite common [2]. Although the health sector is the largest sector in terms of revenue and employment in India, however, the quality of health care services here is miserable and far from customers satisfaction [3]. In this context, Gronross [4] developed the definition of quality for the first time for the service sector. He stated that quality is the consequence of an evaluation process, where the quality users relate their expectations with the perceptions that they received from the product or services. According to Zeithaml et al. [5] service quality is based on customers’ judgments of how services fulfil or exceed their expectations.

Patients demand error-free care, so hospitals usually make an effort to provide services in compliance with objective quality standards. Patients’ expectations, however, may not always be met by these services, indicating that patient expectations are important in determining the quality of the services. Service users these days are well-informed enough to assess quality by contrasting a hospital’s offerings with those of other hospitals. Resultantly, hospitals stand to gain from having a clear understanding of how patients view and anticipate their services [6].

## Review of literature

### Existing models for measuring service quality

The process of measuring service quality is extremely intricate. Researchers have developed several methods over time for evaluating and quantifying the quality of services. Five models have been identified for measuring the quality of healthcare services in the literature, namely, Donabedian’s model of care [7], and the SERVQUAL, SERVPERF, HEALTHQUAL, PubHospQual, HCSQ and HospitalQual models [8]. SERVQUAL is regarded as an all-inclusive metric for evaluating the expected and perceived level of quality of services. The SERVQUAL was developed by Parasuraman et al. [9] who defined service quality in various sectors as the gap between customers’ expectations and perceptions of service quality. According to Gilbert et al. [10] the SERVQUAL model is more in line with identifying patients’ expectations than with what patients have actually experienced when receiving care. The SERVQUAL model has been criticized for failing to show service gaps [11, 12]. According to Cronin et al. [13] the main criticism of the SERVQUAL model is that, rather than only measuring attitudes, it adapts its expectation-disconfirmation model SERVQUAL’s conceptualization and operationalization, as it is now sufficient. Since SQ is solely a patient attitude, critics contest that a performance-based measure is better appropriate for assessing SQ [14]. Several researchers have also been forced to let go of their expectations when evaluating SQ due to the intangible nature of the service. According to Sharma et al. [15, 16] research, the main predictor of customer satisfaction is service performance. With the rapid growth and necessity of hospitals and health care services, it has become vital to understand patients’ expectations of service delivery reliability, responsiveness, assurance, and empathy [12, 17]. This is why SERVQUAL is still the recommended model for assessing SQ in the healthcare sector, despite criticisms in the service quality literature [18]. The final SERVQUAL model comprises of twenty-two paired items covering five dimensions that are used to measure the gap between the expected service quality and the received service quality, and this questionnaire has been widely adopted for explaining consumer perceptions of service quality. The final five dimensions are as follows [19, 20]:

1. Tangibles (physical facilities, equipment and appearance of personnel).
2. Reliability (ability to perform the promised service dependably and accurately).
3. Responsiveness (willingness to help customers and provide prompt service).
4. Assurance (including competence, knowledge and courtesy of employees and their ability to inspire, trust and confidence, credibility and security).
5. Empathy (including access, communication and understanding the customer).

### Rationale of the study

Carman [21], Babakus et al. [22] and Brown et al. [23] stated that in specific service situations, it may be necessary to delete or modify some of the SERVQUAL dimensions or even introduce new ones. Also, these authors demonstrated several shortcomings in the original SERVQUAL scale. Very few studies in India, such as Pramanik [20], have explored the obstacles that hinder service quality in urban and rural hospitals and examined the difference in the quality of healthcare services using existing tools. Tripathi et al. [24] assessed the technical quality of services via SERVQUAL, and data were collected only from healthcare employees. To the best of our knowledge, only one study, Chahal et al. [25], has focused on developing a multidimensional scale for healthcare service quality (HCSQ) in the Indian context. The limitation of this study was that it was performed in only one public hospital in North India. Additionally, the study fails to include/highlight significant regional indicators of service quality in their developed HCSQ model. Keeping this in mind, this study developed and validated the SERV-INDIA model as a tool specifically designed by incorporating regional indicators such as baksheesh which can affect service quality and explore the gap between patients’ expectations and their perceptions [26].

### Research objectives

This research paper is a part of an ongoing doctoral thesis project. Therefore, the primary focus of this particular research segment is to achieve these objectives:

1. To design a comprehensive and more favourable model for measuring service quality in the existing healthcare setting in India.
2. To validate the model for content and reliability as fit for use in healthcare settings.

### Methodology

#### SERV-INDIA tool development process

A sequential mixed-method approach was applied to develop the SERV-INDIA instrument. In this development process, the first step was domain identification process which began with a comprehensive literature review of existing service quality models in healthcare with main focus on studies conducted in developing countries like India, Iran, Bangladesh etc. Carman [21], Babakus et al. [22], Brown et al. [23], and Endeshaw [27] concluded that generic models are no longer sufficient for measuring the quality of healthcare services. Endeshaw [27] also recommended that developing countries should develop their own models for measuring the quality of healthcare services. The lack of indigenous literature led to our derivation of preliminary insights from models developed in other countries. The construction of the SERV-INDIA instrument was an indigenously developed instrument inspired by SERVQUAL, along with a literature synthesis of documents, existing concepts, and theories to obtain quantitative data.

**Fig. 1:**
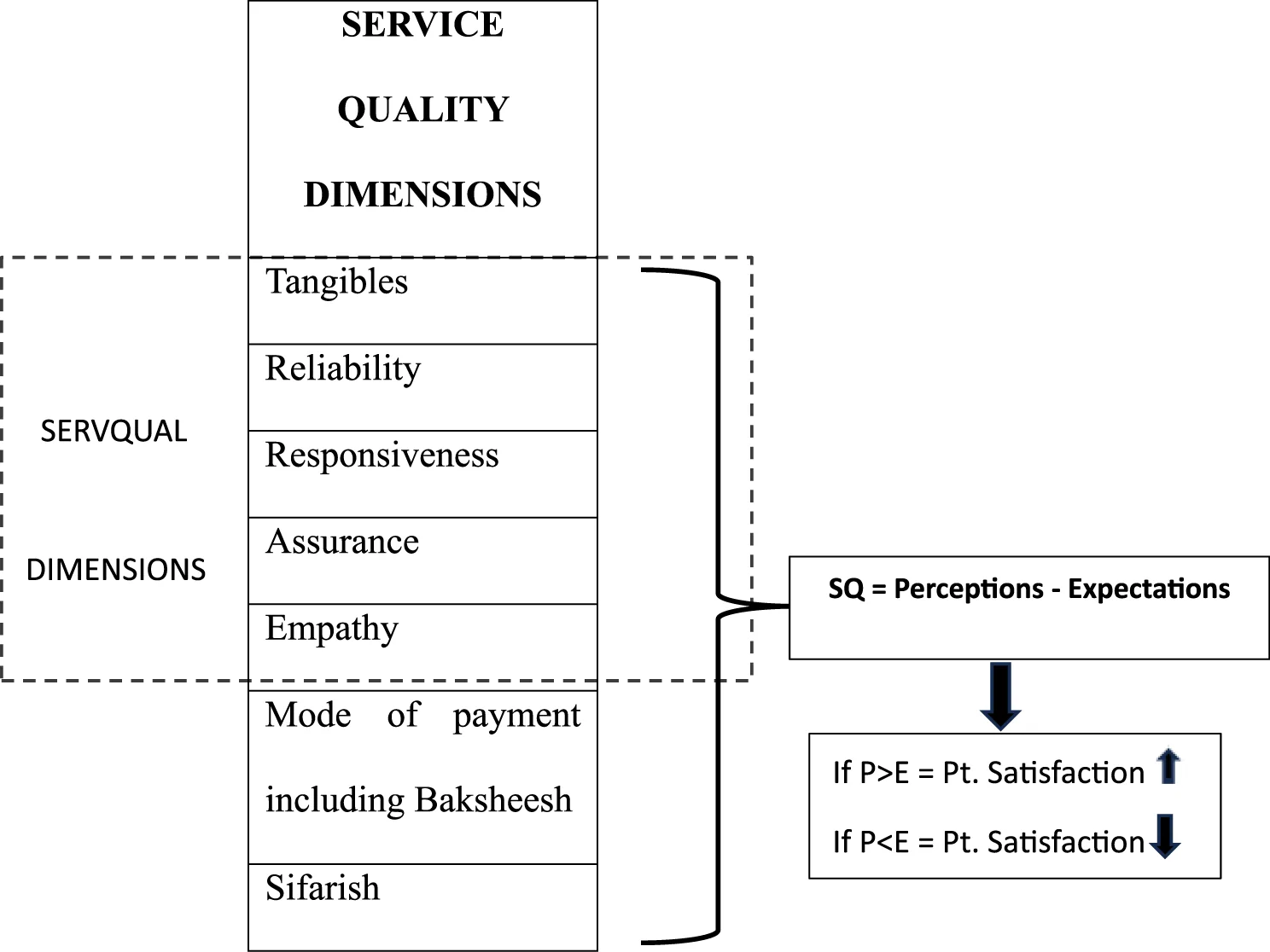
Research model for SERV-INDIA tool

To identify specific factors relevant to the Indian healthcare context, in-depth interviews were conducted with 10 healthcare experts (03 physicians, 04 hospital administrators, and 03 healthcare policy experts). Semi-structured interviews with three focus groups (*n* = 8 participants) were conducted, each representing diverse socioeconomic backgrounds, to identify patient-perceived dimensions of service quality. It became clear from qualitative interviews with experts and focus groups that notions of Sifarish (reference) and baksheesh (extra money for proper service) were seen as impacting how well a service was perceived. Inequity, another declining facet of service pointed out by the experts, is widespread in the country and among people. Overall service satisfaction quickly declines in situations where equity is not being delivered fairly. So, the idea of adding Equity as a dimension was fully supported by healthcare experts as well as the focus groups because of the differences in the care received by patients who had taken treatment under government-aided insurance schemes such as Ayushman Bharat vs. patients paying out of pocket (cash or card) and patients with private insurance. This led to the identification of 07 potential domains for the SERV-INDIA model (see Fig. 1), which includes two regionally specific domains that are “Mode of payment including Baksheesh” and “Sifarish.” Also, the initial item pool consisted of 42 items across these 07 domains.

#### Content validation

Expert involvement was considered necessary because the study involved the development of a new model, i.e., SERV-INDIA tailored to the Indian healthcare context, which required careful review and validation before field use. Five experts were selected who met the selection criteria outlined below to provide diverse yet focused input. The selected expert panel represented key domains relevant to the study, i.e., academics, hospital administration, clinical practice, quality management, and medical statistics (Table 1). Although the experts participated in a professional capacity, they were assigned codes for reporting purposes to maintain consistency and clarity in data presentation.

### Selection criteria for experts

- Minimum 15 years of healthcare sector management experience.
- Active involvement in healthcare research activities.
- Representation from both public and private sectors.
- Demonstrated expertise in healthcare quality improvement.

**Table 1:** Expert panel for the study. Source: Author’s own

Expert Code	Designation	Sector
E1	Former Vice Chancellor, Elite State University of India.	Public
E2	Medical Superintendent with 20 years of Clinical experience	Public
E3	Managing Director of a 100-bedded multispecialty hospital	Private
E4	Quality and Operations Head of a multi-chain hospital group	Private
E5	Retired Senior Statistician of a 3,194-bedded Tertiary Care Public Hospital, India	Public

Experts rated each item based on relevance, clarity, and cultural appropriateness using a 4-point Likert scale. This includes factors like the suitability of the items, tasks, or questions in the particular domain being evaluated and whether the domain covers a broad enough range of items to enable conclusions to be drawn [28]. The Content Validity Index (CVI) was calculated for each item, and items with CVI < 0.90 were revised, and items with CVI < 0.80 were eliminated. This process reduced the item pool to 25 items for Expectation of Service (Part-1) questionnaire and 28 items for Perception of Service (Part-2) questionnaire. Also, to address the potential response bias for culturally sensitive items related to Baksheesh and Sifarish, indirect wording techniques recommended by Fisher [29] for sensitive questions were used. SERV-INDIA was developed using SERVQUAL as a generic base model, in which three sections were adopted with modifications as per the context given by Waiba et al. [30] as follows:

#### Section A

General profile of the demographic details of the respondents, such as gender, age, qualification, and mode of payment.

#### **Section B**

Expectation of Service (Part-1): Twenty-five items on a five-point Likert-type scale of strongly agree, agree, neutral, disagree, and strongly disagree were used.

#### **Section C**

Perception of Service (Part-2): Twenty-five items on a five-point Likert-type scale of strongly agree, agree, neutral, disagree, and strongly disagree were used. An additional three items on the Ranking scale were included to obtain more insights into overall service quality and the satisfaction index.

#### Translation and cultural adaptation

The SERV-INDIA scale was translated from English into Punjabi, following the guidelines by Beaton et al. [31]. Forward translation was performed by a bilingual expert fluent in both English and Punjabi. The translated version was reviewed for clarity, cultural relevance, and comprehensibility among the target population. To ensure semantic and conceptual equivalence, the Punjabi version was subsequently back-translated into English by an independent bilingual expert with English as the native language. The back-translated version was compared with the original English instrument, and minor discrepancies were resolved through consensus to preserve meaning and contextual accuracy. This process ensured linguistic validity and cultural appropriateness of the SERV-INDIA scale for use in the Punjabi-speaking population.

#### Research design and study setting

This study used a cross-sectional quantitative design, with a convenient sampling technique and the data were collected via SERV-INDIA questionnaires. Punjab state of India was selected for this pilot testing study, as the state is renowned for providing the best health-care services in the country. Twenty hospitals in the Punjab region were approached for the study, but only ten hospitals provided written approvals to conduct the study. The questionnaire was pretested three times to ensure that the wording, format, length, and sequencing of questions were appropriate. During each successive pre-test, feedback was obtained from approximately ten hospital users to help refine the quality of the measures [27]. Pilot survey testing was then done in ten multi-speciality hospitals in Punjab, India, and a total of one hundred forty Out-patients (OP) & In-patients (IP) were included. Out of one hundred forty patients, only one hundred five patients participated in both the phases (Part-1 & Part-2) of the study. But after excluding incomplete responses, the final sample consisted of one hundred three participants (*n* = 103). The psychometric properties of the SERV-INDIA instrument were also evaluated using the following measures:

- Internal consistency reliability: Using Cronbach’s alpha and Pearson’s r Correlations for each domain and the overall scale.
- Known-group validity: Comparing service quality perceptions between different payment groups (cash vs. insurance).
- Construct validity: Using Confirmatory Factor Analysis (CFA).

#### Data collection & analysis

Among the one hundred and five survey respondents, two respondents had left more than ten questions unanswered and were excluded from the study. The final pilot sample size was one hundred three, with a 73.5% response rate. *Detailed information on OPD and IPD recruitment*,* participation flow*,* and pilot sample considerations was provided in Supplementary File **1**.* After that, data analysis was performed via STATA 18.0 and SPSS version 22.0 software packages. Data analysis was performed through descriptive statistics, two-sample Wilcoxon rank-sum (Mann–Whitney tests) for testing variations between demographics and to test variations between overall service quality satisfaction levels with different groups of people such as cash patients’ vs. insurance patients, paired t-tests, Confirmatory factor analysis (for evaluating the internal structural validity of the instrument), Wilcoxon signed-rank tests (for testing the significance of the gap between the expectations and perceptions of the patients), Spearman rank correlations (to rank each of the relevant factors of service quality).

### Findings & results

#### Reliability

The survey instrument had an overall excellent level of internal consistency (> 0.95) for both parts of the questionnaire model (Part 1 & Part 2), as shown item-wise in Table 2.

**Table 2:** Reliability analysis (Part 1 & Part 2)

Item Code	Item Description	Item–Total Correlation	Cronbach’s α if Item Deleted
*Part 1 (Expectations of Patients)*			
Q1_E	Up-to-date equipment	0.896	0.953
Q2_E	Visually appealing facilities	0.901	0.953
Q3_E	Clear signage and directions	0.895	0.953
Q4_E	Clean rooms and toilets	0.902	0.953
Q5_E	Front office warm and polite	0.887	0.952
Q6_E	Follow-up reminders	0.871	0.953
Q7_E	Doctors punctual	0.805	0.953
Q8_E	Doctors listen to patients	0.904	0.953
Q9_E	Doctors skilled and knowledgeable	0.883	0.953
Q10_E	Nursing staff punctual	0.789	0.953
Q11_E	Nursing staff skilled and trained	0.835	0.953
Q12_E	Treatment decisions discussed	0.880	0.953
Q13_E	Timely lab reports	0.866	0.953
Q14_E	No unnecessary diagnostics	0.828	0.953
Q15_E	Caring and warm attitude	0.898	0.953
Q16_E	Respect for privacy	0.855	0.953
Q17_E	Understanding discomfort	0.876	0.953
Q18_E	Payment mode affects care	0.425	0.957
Q19_E	Better care under govt insurance	0.677	0.957
Q20_E	Better care under private insurance	0.624	0.957
Q21_E	Better care with out-of-pocket payment	0.676	0.955
Q22_E	Staff expects Baksheesh	0.310	0.959
Q23_E	Better care when tipped	0.367	0.959
Q24_E	Influence helps get better care	0.571	0.957
Q25_E	Influence saves time	0.520	0.958
*Part 2 (Perceptions of Patients)*			
Q1_P	Up-to-date equipment	0.902	0.963
Q2_P	Visually appealing facilities	0.901	0.963
Q3_P	Clear signage and directions	0.895	0.963
Q4_P	Clean rooms and toilets	0.901	0.963
Q5_P	Front office warm and polite	0.896	0.963
Q6_P	Follow-up reminders	0.883	0.963
Q7_P	Doctors punctual	0.801	0.963
Q8_P	Doctors listen to patients	0.881	0.963
Q9_P	Doctors skilled and knowledgeable	0.883	0.963
Q10_P	Nursing staff punctual	0.872	0.963
Q11_P	Nursing staff skilled and trained	0.819	0.963
Q12_P	Treatment decisions discussed	0.853	0.963
Q13_P	Timely lab reports	0.585	0.965
Q14_P	No unnecessary diagnostics	0.392	0.967
Q15_P	Caring and warm attitude	0.891	0.963
Q16_P	Respect for privacy	0.854	0.963
Q17_P	Understanding discomfort	0.870	0.963
Q18_P	Payment mode affects care	0.402	0.966
Q19_P	Better care under govt insurance	0.600	0.965
Q20_P	Better care under private insurance	0.640	0.965
Q21_P	Better care with out-of-pocket payment	0.524	0.966
Q22_P	Staff expects Baksheesh	0.741	0.965
Q23_P	Better care when tipped	0.718	0.965
Q24_P	Influence helps get better care	0.745	0.965
Q25_P	Influence saves time	0.726	0.965
Q26_P	Overall satisfaction with treatment	0.776	0.964
Q27_P	Overall satisfaction with quality of care	0.720	0.964
Q28_P	Likelihood to recommend hospital	0.729	0.964

Overall Cronbach’s α (Expectation scale) = 0.956

Overall Cronbach’s α (Perception scale) = 0.965

Note: Item codes are presented with a concise description was derived from the SERV-INDIA questionnaire. The complete questionnaire is provided as Supplementary File 2

#### Known-group validity

The known-group validity of the survey instrument was checked using a two-sample Wilcoxon rank-sum test (Mann-Whitney U test) as the data was not normally distributed, violating the assumptions of the t-test. When service quality perceptions between different payment groups (cash vs. insurance) were compared, statistically significant variations between different groups of people were seen. Patients who paid out of their pockets were more satisfied than insurance patients (both public and private), with a p-value < 0.0066.

#### Construct validity

The construct validity of the survey instrument was evaluated using Confirmatory factor analysis (CFA) for both Part-1 and Part-2 of the SERV-INDIA questionnaire.

**Table 3:** Confirmatory factor analyses (Part 1 & Part 2)

Fit Index	Value	Acceptable Threshold		Interpretation	
*Part 1 (Expectations of patients)*					
**CFI** (Comparative Fit Index)	0.876	≥ 0.90 (Good), ≥ 0.80 (Acceptable)		Approaching good fit	
**TLI** (Tucker-Lewis Index)	0.836	≥ 0.90 (Good), ≥ 0.80 (Acceptable)		Moderate fit	
**RMSEA** (Root Mean Square Error of Approximation)	0.149	≤ 0.08 (Good), ≤ 0.10 (Acceptable)		Still too high; suggests model misfit	
**SRMR** (Standardized Root Mean Square Residual)	0.036	≤ 0.08 (Good)			Good fit
*Part 2 (Perceptions of patients)*					
**CFI** (Comparative Fit Index)	0.804	≥ 0.90 (Good), ≥ 0.80 (Acceptable)			Acceptable fit
**TLI** (Tucker-Lewis Index)	0.769	≥ 0.90 (Good), ≥ 0.80 (Acceptable)			Approaching acceptable fit; suggests need for modifications if other validity indicators are poor.
**RMSEA** (Root Mean Square Error of Approximation)	0.157	≤ 0.08 (Good), ≤ 0.10 (Acceptable)			High error; poor model fit
**SRMR** (Standardized Root Mean Square Residual)	0.065	≤ 0.08 (Good)			Good fit

The CFA model, as shown in Table 3, demonstrated acceptable fit on multiple indices for both parts of the questionnaire (CFI = 0.876 & 0.804, TLI = 0.836 & 0.769, SRMR = 0.036 & 0.065) except the RMSEA value (0.149 & 0.157), suggesting poor model fit. But the Root Mean Square Error of Approximation (RMSEA) has been known to be sensitive to sample size, particularly in studies with less than 200 participants, like Kenny et al. [32] & Chen et al. [33] also demonstrated that RMSEA tends to over-reject correctly specified models with small sample sizes. Similarly, Herzog et al. [34], stated that in sample sizes under 200, RMSEA often exhibits positive bias. The model demonstrates conceptual integrity across multiple domains, like reliability testing, face validity by experts, known group validity, and also CFI, SRMR & TLI were acceptable, suggesting that the high RMSEA value likely represents a statistical artifact of sample size rather than the model misfit. Future research with larger samples will provide opportunities to further validate these findings clearly.

##### **Participants’ demographic distribution**

Table 4 gives a summarised description of the demographic distribution of the 103 patients who participated in the study, both Part 1 & Part 2.

**Table 4:** Demographic distribution (*n* = 103)

**Gender**	**Frequency**	**Percentage**
Male	53	51.46
Female	50	48.54
**Age**		
Under 25 years	24	23.30
25–35 years	26	25.24
35–45 years	35	33.98
Above 46	18	17.48
Level of Education		
Primary	35	33.98
High School	50	48.54
Graduate and above	18	17.48
Mode of Payment		
Cash (Out of Pocket)	43	41.75
Private Insurance	27	26.21
Government Insurance	33	32.04
Registration Type		
Outpatient Department (OPD)	55	53.40
Inpatient Department (IPD)	48	46.60
How do you know about us		
Word of mouth	1	0.97
Referral	2	1.94
TV/Newspaper/Radio	34	33.01
Search Engine	2	1.94
Specialists	9	8.74
Approachable	48	46. 60
Others, pl specify	7	6.80

### Results based on demographics

The demographic profile of the respondents is displayed in Table 4. The proportion of male and female respondents was 51.4% and 48.5%, respectively. The majority of respondents (52.1%) were in the age group of 30 to 40, while those aged above 46 constituted a small proportion (17.4%) of respondents. Regarding their education level, the majority of the respondents (48.5%) have a basic high school education. This means that the level of education among respondents of the study was average. Despite their basic education level, 58.2% of respondents were found using health insurance as their mode of payment for treatment, whether public or private, while 41.7% were found paying out of their pockets. Also, the majority of the patients (46.6%) who visited the hospital had chosen the respective hospital because the hospital was accessible to them. A two-sample Wilcoxon rank-sum (Mann–Whitney) test was used to test demographics against overall service quality satisfaction levels. No statistically significant results were seen when overall service quality satisfaction levels were compared across demographics like age, gender, level of education, and registration type. But statistically significant variations between different groups of people paying via different modes of payment, such as cash patients and insurance patients, were seen. Patients who paid out of their pockets were more satisfied than insurance patients (both public and private), with a p-value < 0.0066.

### Results based on Patients’ expectations and perceptions of service quality (Part-1 & Part-2)

The results indicated that participants had higher levels of expectations for all variables, with mean scores ranging from 4.5 to 3.4, with ‘Infrastructure variables’ being highly ranked and ‘Sifarish’ being the lowest ranked variable. The perceptions scale results were also similar to the expectations scores, indicating that participants had higher levels of perceptions, with mean scores ranging from 4.5 to 3.5, with ‘Infrastructure variables’ being highly ranked and ‘Sifarish’ being the lowest ranked variable, as shown in Table 5.

**Table 5:** Patients’ expectations & perceptions of service quality (Part-1 & Part-2)

Variable	Mean	Std error	Std. dev.	Rank
Part 1 (Patients Expectations of Service Quality)				
Infrastructure_E	4.59	0.08	0.84	1
Front Office_E	4.56	0.09	0.94	2
Caregivers_E	4.50	0.08	0.82	3
Diagnostics_E	4.48	0.08	0.83	4
Mode of payment_E	3.64	0.09	0.93	5
Sifarish_E	3.33	0.13	1.33	6
Part 2 (Patients Perceptions of Service Quality)				
Variable	Mean	Std error	Std. dev.	Rank
Infrastructure_P	4.58	0.08	0.85	1
Front Office_P	4.54	0.09	0.95	2
Caregivers_P	4.48	0.08	0.84	3
Diagnostics_P	4.35	0.07	0.80	4
Mode of payment_P	3.93	0.11	1.16	5
Sifarish_P	3.51	0.16	1.68	6

### Variations between patients’ expectations and their perceptions of service quality

Table 6 shows the variations between patients’ expectations of services and their perceptions of the quality of service received or offered by hospitals. This relationship was explored via the paired samples t-test procedure. The results revealed a statistically significant difference (P value < 0.001) between patients’ perceptions of the quality of service in hospitals and patients’ expectations for domains such as Diagnostics, Mode of Payment and Sifarish of the SERV-INDIA model, indicating that patients’ expectations regarding diagnostic services were greater than patients’ perceptions, with a mean difference as -0.12, whereas patients’ perceptions regarding modes of payment, including Baksheesh and Sifarish, were greater than patients’ expectations, with mean differences of 0.28 and 0.17, respectively.

**Table 6:** Variations between patients’ expectations and their perceptions of service quality

Paired t-test differences								
				[95% confidence interval]				
Variables	Mean	Std. error	Std. dev.	lower	upper	t	df	*p*-value
Infrastructure	-0.01	0.01	0.14	-0.04	0.01	-1	102	0.31
Front office	-0.01	0.01	0.19	-0.05	0.01	-1	102	0.31
Caregivers	-0.01	0.02	0.26	-0.06	0.03	-0.49	102	0.62
Diagnostics	-0.12	0.02	0.26	-0.18	-0.07	-4.89	102	0.00
Mode of payment including Baksheesh	0.28	0.04	0.491	0.19	0.38	5.92	102	0.00
Sifarish	0.17	0.08	0.868	0.00	0.34	2.09	102	0.03

To better understand the results, the items in the questionnaires were grouped according to the generic SERVQUAL scale dimensions, namely Tangibles, Reliability, Responsiveness, Assurance, and Empathy with the newly introduced variables namely Mode of payment including Baksheesh and Sifarish which were kept the same as shown in Table 7.

**Table 7:** Grouping of items as per SERVQUAL with newly added variables

Dimensions	Items in the questionnaires
Tangible	1,2,4
Reliability	7,10,9,11,13
Responsiveness	3,6,12
Assurance	5,14,16
Empathy	8,15,17
Mode of payment including Baksheesh	18, 19, 20, 21, 22, 23
Sifarish	24, 25

The Wilcoxon signed-rank test was then used to test the significant gap between the expectations and perceptions of the patients. Statistically significant gaps were observed in terms of reliability, assurance and mode of payment including baksheesh dimensions with p-values of 0.0009, 0.0039 and 0.00, respectively. The results revealed that factors such as the on-time delivery of diagnostic tests in the reliability dimension, the ordering of necessary tests and procedures in the assurance dimension and employees asking for Baksheesh (tips for service) in the mode of payment dimension were more statistically affected. Statistically significant correlations were also found when the satisfaction level regarding service quality was compared with that of Baksheesh and Sifarish from the perception results (p-value < 0.001) via the Spearman rank correlation test.

## Discussions

This study successfully developed and tested a model that integrates generic service quality dimensions with culturally relevant factors that had not been studied previously in existing frameworks such as SERVQUAL, SERVPERF, and HOSPQUAL etc. The regional factors integrated into our model exhibited excellent reliability and validity metrics, indicating that the framework is effectively capturing the nuanced realities existing in the Indian healthcare sector. These findings validated the significance of adding region-specific indicators instead of merely adopting international frameworks without any modifications. These findings were consistent with studies like Carman [21], Babakus et al. [22], Brown et al. [23], and Endeshaw [27]. The SERV-INDIA model of service quality was designed with the objective to measure the service quality by examining the variations between expectations and perceptions of patients, specifically keeping in mind the existing Indian healthcare sector. The variation gap between the expectations and perceptions scores was statistically significant for dimensions such as reliability, assurance, mode of payment including Baksheesh. These results were consistent with studies like A’aqoulah et al. [6], Nadi et al. [35], and Tomes et al. [36]. In earlier investigations, researchers looked at the impact of demographic factors in an attempt to determine why expectations were always higher. In this study, these factors had no discernible impact on the mean scores for any of the SERV-INDIA model domains. This finding contrasts with the findings of Li et al. [37] and Zun et al. [38] who reported that demographic factors such as age, education level, registration type: out-patients or in-patients (OPD/IPD) etc. had an impact on patient satisfaction. Additionally, in this study, cash patients who paid out of their pockets were more satisfied than insurance patients (both public and private). Baksheesh (tips for service) from the mode of payment dimension and Sifarish (putting in a word), while also significant, had a direct effect on patient satisfaction. Even though this revelation may appear unanticipated, some thought reveals that it makes sense. Baksheesh and Sifarish now in the present healthcare scenario, promoting increased customer satisfaction; however, as annoyances, these may have only nuisance value. Baksheesh and Sifarish were seen as significantly important to patients before they selected the hospital, to save their time, obtain good quality care and increase their level of satisfaction. This finding contrasts with the findings of Andaleeb [26] who found that Baksheesh was an important indicator of decreased patient satisfaction and viewed it as harassment.

The information presented in this paper is insightful for researchers and practitioners who may want to use the findings to increase patient satisfaction and service quality in Indian hospitals. The SERV-INDIA model identified several service dimensions that hospital patients in developing nations find significant. However, investigations are needed to substantiate and improve the model. In the future, implementing patient-driven quality standards either through more validation of this model or through the identification of new service factors should be viable to help service providers better meet the demands of their patients [26].

### Limitations

This study has certain limitations, even though it is one of the few that has designed a new tool named SERV-INDIA to assess patients’ expectations and perceptions of service quality for the Indian health sector. First, the model development was pilot tested and validated on a smaller population, so the model can be tested on a larger sample population to probe the generalizability of the tool. Second, while this SERV-INDIA model testing was restricted to only one country, i.e., India, with the addition of only a few new cultural dimensions, therefore, it can be replicated or modified for other cultural contexts or developing nations. Nevertheless, in developed economies as well, the situation can be analysed to compare the discrepancies that already exist.

## Conclusion and future work

The main objective of this research was to validate the ‘SERVINDIA’ model, following the methodology of Parasuraman et al. [9], a model that can evaluate healthcare service quality. This study successfully developed and validated a comprehensive service quality assessment model tailored to the Indian healthcare sector. The newly introduced parameters in the scale, were the mode of payment, including Baksheesh and Sifarish, played a crucial role in moulding the perceptions of the patients regarding service quality and, ultimately, the satisfaction index of the patients. There were additional findings and preliminary evidences seen as of the scale’s utility in this pilot study, i.e., when examined the difference between patients’ expectations of service quality and their perceptions of what services they were actually receiving in hospitals. This model also supported the importance of evaluating patient expectations before designing the service delivery process of the hospital because there exists a statistically significant gap between expectation and perception scores on various dimensions. The future direction would be to use this model on a larger sample population across broader age groups, literacy levels, and healthcare organisations so that the generalizability of the tool can be probed.

## Supplementary Information

Below is the link to the electronic supplementary material.

Supplementary Material 1

Supplementary Material 2

## Data Availability

All data generated or analysed during this study were included in this manuscript as supplementary files.
