# Supplementary material for: SERV-India instrument for measuring service quality in healthcare: development and pilot testing
Source: BMC Health Serv Res. 2026 Jun 17;26:1192. doi: 10.1186/s12913-026-14936-x (PMC13520184; doi:10.1186/s12913-026-14936-x)
Supplement: Supplementary file 1 [file 12913_2026_14936_MOESM1_ESM.pdf]

1.1 Pilot Study Sample Size:

Before administering the questionnaire in the main study, a pilot study was conducted to assess the clarity, relevance, and reliability of the SERV-INDIA questionnaire. The flow chart as follows, clearly describes how the sample size was calculated for the pilot study.

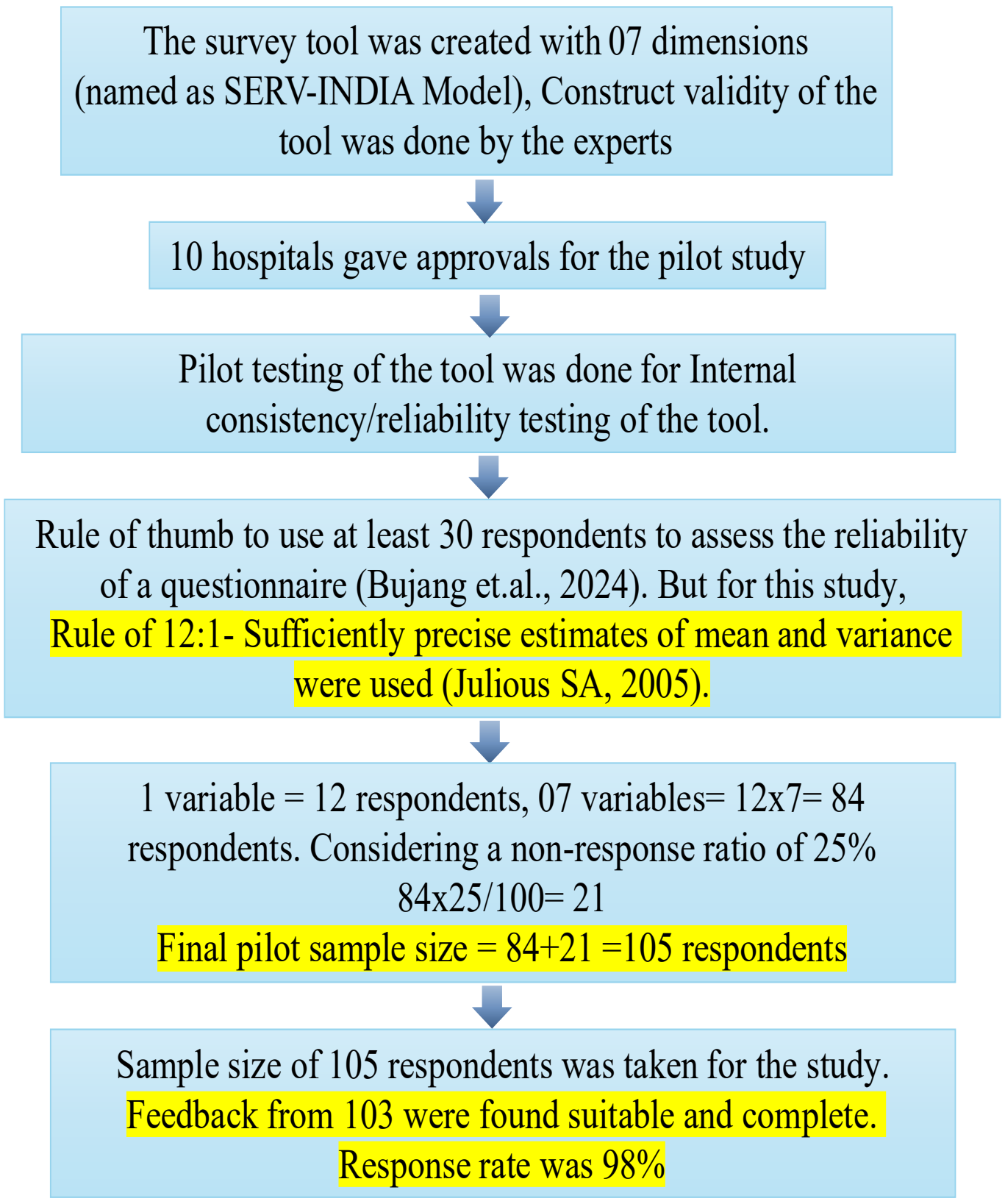

1.2 Inclusion and Exclusion Criteria

The study employed convenience sampling appropriate for pilot instrument development. Eligible participants included adult OPD and IPD patients who had received healthcare services during the study period.

Inclusion Criteria:

- Patients visiting all 10 selected hospitals (OPD and IPD)
- Patients completing both parts of the survey

Exclusion Criteria:

- Critical/non-willing patients
- Patients who left hospital premises before survey completion
- Incomplete survey responses
