# Supplementary material for: SERV-India instrument for measuring service quality in healthcare: development and pilot testing
Source: BMC Health Serv Res. 2026 Jun 17;26:1192. doi: 10.1186/s12913-026-14936-x (PMC13520184; doi:10.1186/s12913-026-14936-x)
Supplement: Supplementary file 2 [file 12913_2026_14936_MOESM2_ESM.pdf]

SERV-INDIA Model

Hospital Type (Ss / Ms): ___

Note: The questionnaire is divided in 3 parts: General Demographic details, Part-1 Measuring Expectations & Part-2 Measuring Perceptions (Actual state of affairs)

Demographic Details:

Gender: Male/Female

Age: Under 25 years/ 25-35 years/ 35-45 years/ Above 46

Level of education: Primary/High school/Graduate & above

Payment Mode: Cash/Pvt. Insurance/Govt. Insurance

Registration Type: OPD/IPD

Part-1 Measuring Expectations

S. No.		Items	Strongly Agree	Agree	Neutral	Disagree	Strongly disagree
1.	**Premises/Infrastructure**	The hospital should have up-to-date equipment					
2.		The hospital's building and physical facilities should be visually appealing					
3.		Signages/Directions should be displayed properly with in the hospital premises.					
4.		The hospital's rooms and toilets should be clean.					
5.	**Front office**	Front office staff should always be warm and polite.					
6.		The staff should always remind you of your upcoming visits. (follow ups, routine check-ups)					
7.		The doctors should be punctual at all times.					
8.		The doctors should listen to what you have to say.					

Hospital Type (Ss / Ms): ___

9.	Caregivers	The doctors should be skilled and knowledgeable.					
10.		The nursing staff should always be punctual at all times.					
11.		The nursing staff appointed by the hospital should be skilled and trained.					
12.		All decisions regarding the treatment and care should be discussed with you.					
13.	Diagnostics	The lab tests and other reports should be delivered punctually.					
14.		Unnecessary tests and diagnostics procedures should never be ordered.					
15.		Do you think employees' attitude towards you will always be caring and warm.					
16.		The staff should always respect your privacy during procedures.					
17.		The staff should understand you if you're feeling discomfort.					
18.	Mode of payment	You expect that mode of payment will affect hospital treatment and care imparted.					
19.		Do you expect that treatment will be better under govt. Insurance schemes like Ayushman Bharat.					
20.		Do you expect that treatment will be better under private insurance schemes.					
21.		Do you expect that treatment will be better when payment is done Out-of-pocket by the patient.					
22.		Do you expect that employees will ask for Baksheesh (tips) for their services.<br><br>If yes, Category of staff					

Hospital Type (Ss / Ms): ___

		________________________					
23.		Do you expect that staff will give good quality care when tipped.					
24.	Sifarish	Do you think that putting in a word/ knowing someone in this hospital helps in getting good quality care.					
25.		Do you think that knowing someone in this hospital saves time.					

Part-2 Measuring Perceptions (Actual State of Affairs)

S. No.		Items	Strongly Agree	Agree	Neutral	Disagree	Strongly disagree
1.	Premises/Infrastructure	The hospital has up-to-date equipment					
2.		The hospital's building and physical facilities are visually appealing					
3.		Signages/Directions are displayed properly with in the hospital premises.					
4.		The hospital's rooms and toilets are clean.					
5.	Front office	Front office staff is warm and polite.					
6.		The staff always reminds you of your upcoming visits. (follow ups, routine check-ups)					
7.		The doctors are punctual at all times.					
8.		The doctors listen to what you have to say.					

SERV-INDIA Model

Hospital Type (Ss / Ms): ___

9.	**Caregivers**	The doctors are very skilled and knowledgeable.					
10.		The nursing staff is punctual at all times.					
11.		The nursing staff appointed by the hospital are highly skilled and trained.					
12.		All decisions regarding the treatment and care are being discussed with you.					
13.	**Diagnostics**	The lab tests and other reports are delivered punctually.					
14.		Unnecessary tests and diagnostics procedures are never ordered.					
15.		Employees' attitude towards you is caring and warm.					
16.		The staff always respect your privacy during procedures.					
17.		The staff understands you if you're feeling discomfort.					
18.	**Mode of Payment**	Mode of payment affects hospital treatment and care imparted.					
19.		In your experience, Treatment is better under govt. Insurance schemes like Ayushman Bharat.					
20.		In your experience, Treatment is better under private insurance schemes.					
21.		In your experience, Treatment is better when payment is done Out-of-pocket by the patient.					
22.		In your experience, do staff expects Baksheesh (tips) for their services.<br><br>If yes, Category of staff<br><br>___________________________					

Hospital Type (Ss / Ms): ___

23.		Staff gives good quality care when tipped.					
24.	**Sifarish**	Putting in a word/ knowing someone in hospital helps in getting good quality care.					
25.		Knowing someone in hospital saves time.					

Q26. Overall, how satisfied are you with the treatment given in this hospital:

Not satisfied			Very Satisfied
1	2	3	4

Q27. Also, how satisfied are you with the quality of care given in this hospital:

Not satisfied			Very Satisfied
1	2	3	4

Q28. How likely you are going to recommend this hospital to others:

Not likely			Very Likely
1	2	3	4

Any other information/ experience you want to share:

____

____

____
